# Genome-Wide Alteration of Histone H3K9 Acetylation Pattern in Mouse Offspring Prenatally Exposed to Arsenic

**DOI:** 10.1371/journal.pone.0053478

**Published:** 2013-02-06

**Authors:** Andrea A. Cronican, Nicholas F. Fitz, Alexis Carter, Muzamil Saleem, Sruti Shiva, Aaron Barchowsky, Radosveta Koldamova, Jonathan Schug, Iliya Lefterov

**Affiliations:** 1 Department of Environmental and Occupational Health, University of Pittsburgh, Pittsburgh, Pennsylvania, United States of America; 2 Department of Pharmacology and Chemical Biology, University of Pittsburgh, Pittsburgh, Pennsylvania, United States of America; 3 Functional Genomics and Next-Generation Sequencing Core, Perelman School of Medicine, University of Pennsylvania, Philadelphia, Pennsylvania, United States of America; University of Kansas Medical Center, United States of America

## Abstract

Chronic exposure to arsenic in drinking water, especially *in utero* or perinatal exposure, can initiate neurological and cognitive dysfunction, as well as memory impairment. Several epidemiological studies have demonstrated cognitive and learning deficits in children with early exposure to low to moderate levels of arsenic, but pathogenic mechanisms or etiology for these deficits are poorly understood. Since *in vivo* studies show a role for histone acetylation in cognitive performance and memory formation, we examined if prenatal exposure to arsenic causes changes in the epigenomic landscape. We exposed C57Bl6/J mice to 100 μg/L arsenic in the drinking water starting 1 week before conception till birth and applied chromatin immunoprecipitation followed by high-throughput massive parallel sequencing (ChIP-seq) to evaluate H3K9 acetylation pattern in the offspring of exposed and control mice. Arsenic exposure during embryonic life caused global hypo-acetylation at H3K9 and changes in functional annotation with highly significant representation of Krüppel associated box (KRAB) transcription factors in brain samples from exposed pups. We also found that arsenic exposure of adult mice impaired spatial and episodic memory, as well as fear conditioning performance. This is the first study to demonstrate: a) genome wide changes in H3K9 acetylation pattern in an offspring prenatally exposed to arsenic, and b) a connection between moderate arsenic exposure and cognitive impairment in adult mice. The results also emphasize the applicability of Next Generation Sequencing methodology in studies aiming to reveal the role of environmental factors, other than dietary restriction, in developmental reprogramming through histone modifications during embryonic development.

## Introduction

Chronic human exposure to arsenic through drinking water is a global public health concern that may affect as many as 130 million people daily. Arsenic leaching from rock into aquifers and surface waters is the primary source of exposure, with reports of 6–21% of US wells containing arsenic in excess of the EPA safe drinking water standard of 10 μg/L [1], [2]. Local exposures can be much higher depending on rock variations, such as the 17% of wells in excess of 100 μg/L found in counties in the western part of the US and 10% of wells in excess of 500 μg/L found in Bangladesh [3], [4]. In addition to the finding that these exposures cause cancer in different organs and significant mortality from cardiovascular and respiratory diseases [5], arsenic exposure has been associated with a number of developmental neurological disorders, peripheral neuropathies, and neuromuscular dysfunction. While neuropathies and some sensorimotor deficits have been attributed to high levels of arsenic impairing ATP generation and promoting necrosis, mechanisms involved in neurological deficits caused by chronic low to moderate arsenic exposure are not well understood [3].

Epidemiological studies have correlated arsenic exposure to various learning deficits and cognitive impairment in children in Bangladesh [6], West Bengal [7], and Mexico [8], [9], as well as in adults in Texas, USA [10]. Despite clear evidence that arsenic is a developmental neurotoxicant, the molecular mechanisms for the increased risk of cognitive and memory impairment remains unclear. A number of animal studies have focused on explaining neurotoxic effects of arsenic from prenatal exposure, as well as exposure in young adults. However, some may be questionable due to exposures greatly exceeding possible human exposures. In rats, prenatal and early life exposure to 100 mg/L arsenic in drinking water decreased neuromotor reflexes and produced deficits in learning [11]. Studies in adult rats fed 20 mg/kg of arsenic in chow or exposed to 68 mg/L of the toxicant in water, demonstrated impaired learning and memory, changes in dopamine levels and alteration of ultra-structural brain morphology [12], [13]. A study in mice that used human relevant perinatal exposures (55 μg/L), demonstrated arsenic increased learned helplessness and reduced performance in forced swim tests [14]. In addition, *in utero* exposure to 50 μg/L impaired learning and memory of adult offspring [15]. However, few molecular details have been provided to explain the pathogenic mechanisms resulting from *in utero* or adult exposure to arsenic.

There is currently an intense focus on epigenetic regulation of phenotypes to identify chronic enhancement of disease risk resulting from arsenic exposure during discrete developmental windows. Arsenic is the only environmental toxicant that causes changes in all three epigenetic markers – DNA methylation, histone modifications and expression of noncoding RNAs [16]. Since arsenic is extensively methylated during its metabolism (the source of methyl groups being S-adenosylmethionine, SAM), numerous studies have addressed changes in DNA and histone methylation with confounding results [17]. Interestingly, in a report on global changes in histone modifications, a decreased acetylation of H3K9 was observed in peripheral mononuclear cells of workers exposed to arsenic [18]. The link, however, between changes in epigenetic signals from arsenic exposure and changes in phenotypes linked to disease later in life are not well defined. In this report we present the results of a study undertaken to reveal arsenic-induced changes in enrichment of epigenetic marks in brain samples of offspring with *in utero* arsenic exposures. Chromatin immunoprecipitation followed by massive parallel sequencing (ChIP-seq) using an antibody against acetylated lysine 9 of histone 3 (H3K9Ac) was applied to evaluate the differences in H3K9 acetylation pattern genome-wide and to compare Gene Ontology terms and functional annotations of affected genes between exposed and control pups. We also present the results of behavioral testing conducted with young adult C57BL/6J mice exposed to human-relevant levels of arsenic.

## Results

### Birth outcomes

**Figure 1:**
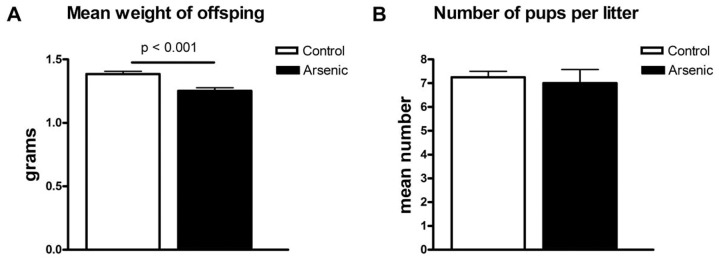
Pups' weight but not litter size is decreased by prenatal exposure to arsenic. **A**. The mean weight of the pups is decreased approximately by 10% in arsenic treated mothers. P<0.05 by t-test. **B**. Number of pups per pregnant dam is not significantly changed by arsenic treatment.

For prenatal exposure female mice (8–9 weeks old) were initially provided with either 100 μg/L arsenic in spring water (arsenic exposed) or plain spring water (control) for 1 week. Males were then added to the cage and removed 8 days after 1st introduction. Females continued under exposure for the duration of their pregnancy. Pups were collected within 24 hrs of birth. Although differences in litter size were not observed and there were no visible gross anatomical changes of P1 pups, there was a significant difference between the average individual birth weight of arsenic exposed (1.25±0.02 grams) and control (1.38±0.02 grams) offspring (Figure 1).

### Genome-wide mapping of H3K9 acetylation in offspring prenatally exposed to arsenic

The cortices and hippocampi of each pup were dissected and tissue from each litter was pooled and used for ChIP. ChIP-seq libraries were generated as described in Materials and Methods and sequenced on Illumina hiSeq2000 instrument to generate approx. 24 million total reads from DNA samples of cortices and hippocampi of exposed pups. Parallel sequencing of input DNA resulted in approx. 62 million total reads. Similarly ChIP-Seq of unexposed pups (Control) yielded approx. 29 million total reads, with approx. 28 million total input reads. The sequence tags then were aligned to the mouse genome (mm9). We used the peak calling algorithm HOMER to evaluate fold change over input and peak height, as well as to identify significant regions of enrichment and calculate a false discovery rate (FDR). This was followed by profile smoothing and splitting to identify individual peaks. In both conditions (arsenic exposure and control) effective sequencing reads (tags) were processed by the algorithm for identification of regions of enrichment at stringent predetermined FDR of 0.1%. High quality peaks - regions of H3K9Ac significant enrichment compared to the input values were connected to genes at a distance of up to 100KB from the transcription start site (TSS). This filtering step reduced the number of peaks/regions of enrichment subjected to analysis in the control and arsenic exposed samples to 37,946 and 8,577 respectively.

#### Prenatal Exposure to arsenic results in genome-wide hypo-acetylation at H3K9

**Figure 2:**
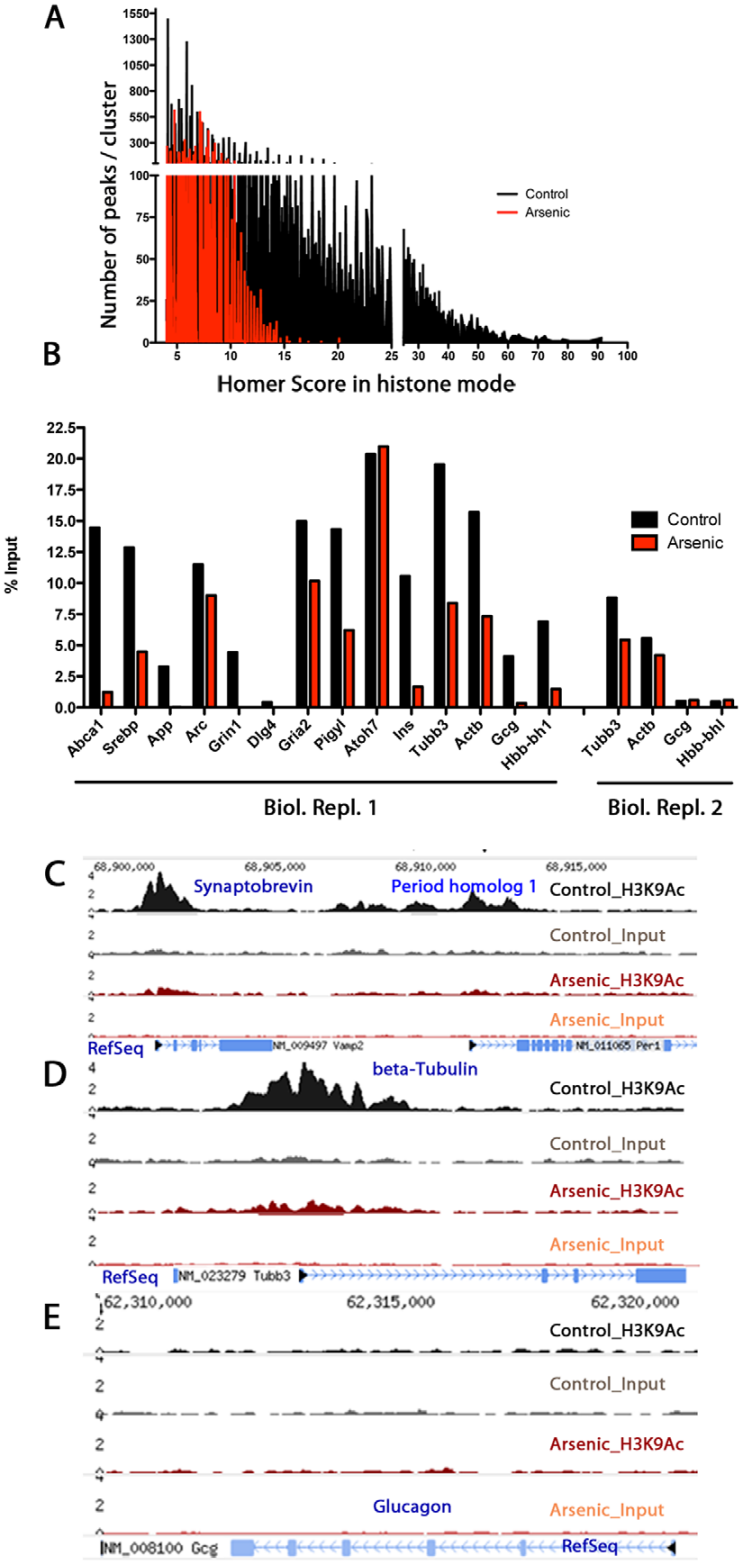
Global hypoacetylation at H3K9 in response to arsenic exposure during embryonic life. Each of the clusters on the histogram in Panel **A** represent all significant peaks of enrichment with the same HOMER score from arsenic (in **red** 6.71 median, 6.86 average, 4.03 min, 20.13 max, 8577 total peaks) and control (in **black:** 9.07 median, 11.70 average, 4.02 min, 91.62 max, 37946 total peaks) treated samples. **B**. ChIP-QPCR validation assays from two biological replicates demonstrate differences in H3K9Ac enrichment in the proximal promoters of randomly selected genes from arsenic and control treated samples. Abbreviations: Abca1, ATP-binding cassette, sub-family A, member 1; Srebp, Sterol regulatory element binding transcription factor 1; App, Amyloid precursor protein; Arc, activity regulated cytoskeletal-associated protein; Grin1, Glutamate receptor, ionotropic, N-methyl D-aspartate 1; Dlg4, Discs, large homolog 4; Gria2, Glutamate receptor, ionotropic; Plgyl, Phosphatidylinositol glycan anchor biosynthesis, class Y-like; Atoh7, Atonal homolog 7; Ins, Insulin; Tubb3, β-Tubulin; Actb, β-Actin; Gcg, Glucagon; Hbb-bh1, Hemoglobin Z, beta-like embryonic chain. **C, D, E**. Screen shots taken from gene browser TessLA (http://ngsc.med.upenn.edu), demonstrate the difference between the levels of H3K9Ac enrichment in the proximal promoters of several genes. Genes, used throughout the study as positive - Tubb3 (expected enrichment), and negative - Gcg (no enrichment) controls for quality validation of the ChIP samples, as well as some randomly selected genes (Vamp2 and Per1) are shown.

We created a histogram of the HOMER values for peaks from control and arsenic-exposed H3K9ac-enriched regions. In addition to the difference in the total number of peaks, the graph in Figure 2, panel A, demonstrates that HOMER scores from the arsenic sample (shown in red) are significantly lower, with the majority of the regions clustered around a score of 6.7. In fact, in the arsenic exposure ChIP-seq dataset there were no peaks with a HOMER score above 20.13. In contrast, the number of peaks from the control sample (in black) at each of the clusters was higher with a significant number of peaks scored above 10 and up to 91.67. Screen shots taken from the genome browser TessLA (http://ngsc.med.upenn.edu), also demonstrate the difference between the levels of H3K9Ac enrichment in the proximal promoters of several genes (Figure 2, panel C). To further confirm the differences in global acetylation, we calculated and compared the enrichment of H3K9Ac as % of input using primers in the proximal promoters of several genes (see Figure 2, panel B). The results of ChIP-QPCR assays performed on ChIP DNA from each of the conditions demonstrate a difference in acetylation at H3K9 for the positive (Tubb3 and Act-b) and negative (Gcg and hbb-bh1) control genes. The assays have been repeated and results confirmed with ChIP DNA from two biological replicates.

### Functional pathway analysis

To identify biological processes in which the submitted genes were involved, Gene Ontology (GO) analysis was performed using the Database for Annotation, Visualization and Integrated Discovery (DAVID, version 6.7; [19], [20]) with mm9 RefSeq mRNA annotations and the entire mouse genome as a background model. The GO analysis was performed for the top 3000 regions with a distance to the nearest gene ≤1 kb. For GO analysis, we focused on the promoter regions with the highest HOMER scores that represent the genes with a high level of H3K9 acetylation and thus assumed to be in a transcriptionally active state. Fold enrichment score in the output tables was provided by DAVID as in the functional annotation charts, and is defined as % genes in a given class divided by genes in genome in the same class. For example enrichment of 10 would equal 10% of user genes vs. 1% of genes in genome. Clusters were considered significant at fold enrichment ≥1.5 and Benjamini factor ≤0.05. Although arsenic exposed samples had less acetylation globally, multiple clusters were similarly enriched in the gene lists generated for both control and arsenic samples (Tables 1 and 2). The analysis, however, also demonstrates that arsenic exposure results in an increase in zinc finger transcription factor class, with an emphasis on Krüppel associated box (KRAB) transcription factors. The KRAB domain is a transcriptional repression domain commonly found in zinc-finger transcription factors. While the absolute level of H3K9 acetylation in the promoter regions of those genes is not increased in arsenic exposed mice compared to control, the relative enrichment is greater. Although it is difficult to provide a mechanistic explanation, the data clearly demonstrate that more KRAB domain containing genes were associated with the 3000 most enriched H3K9Ac peaks in arsenic exposed mice then those in the control samples (see Table 3 for a list of KRAB genes in each condition).

**Table 1:** Unique functional annotation clusters of genes from arsenic and control datasets.

Treatment	Term	Significance	Fold
Arsenic	Molecular Function	(Benjamini)	Enrichment
	Kinase activator	3.09E-02	2.903
	Chromatin/chromatin-binding protein	2.16E-02	2.320
	Microtubule family cytoskeletal protein	4.84E-02	1.770
	KRAB box transcription factor	6.67E-03	1.635
	Zinc finger transcription factor	2.46E-04	1.603
	**Biological Process**		
	NA		
**Control**	**Molecular Function**		
	Non-motor microtubule binding protein	2.44E-02	2.642
	mRNA splicing factor	6.77E-03	2.556
	Ubiquitin-protein ligase	1.91E-03	2.118
	Chaperone	1.25E-02	2.042
	Protein phosphatase	3.07E-02	2.001
	Transcription cofactor	2.63E-02	1.928
	Small GTPase	2.24E-02	1.861
	Ligase	8.72E-04	1.829
	Non-receptor serine/threonine protein kinase	4.78E-03	1.801
	G-protein	1.53E-02	1.752
	Kinase	2.12E-04	1.683
	Protein kinase	1.83E-03	1.667
	G-protein modulator	2.31E-02	1.598
	**Biological Process**		
	Tumor suppressor	2.26E-02	2.447
	DNA repair	1.24E-02	2.115
	General vesicle transport	3.11E-04	2.107
	Protein targeting and localization	3.46E-03	2.071
	Chromatin packaging and remodeling	1.18E-02	2.004
	Protein folding	3.14E-02	1.973
	Protein phosphorylation	6.35E-06	1.758
	Cell cycle control	6.44E-03	1.660
	Protein modification	4.26E-06	1.563
	Apoptosis	2.33E-02	1.502

For category ranking and analysis of biological terms in DAVID, the first 3000 regions of enrichment were filtered by absolute distance of ≤1 kb from the TSS. The final gene lists submitted for analysis consisted of 1757 and 1803 official gene symbols in the arsenic and control groups, respectively. For each of the lists unique Molecular Function and Biological Process terms are generated by satisfying two criteria - fold enrichment >1.5 and statistically significant Benjamini factor <0.05. Note, that in the gene list from arsenic exposed group there are no unique Biological Processes.

**Table 2:** Functional annotation clusters in common between gene lists from arsenic and control datasets.

Arsenic			Control	
Significance	Fold	Term	Significance	Fold
(Benjamini)	Enrichment		(Benjamini)	Enrichment
		**Molecular Function**		
2.29E-02	2.230	mRNA processing factor	4.66E-03	2.353
1.98E-02	2.153	Kinase modulator	1.21E-02	2.094
2.99E-02	1.765	Phosphatase	8.18E-03	1.835
		**Biological Process**		
1.01E-02	2.532	Chromosome segregation	3.42E-02	2.248
1.04E-04	2.046	Mitosis	3.70E-03	1.791
1.15E-02	1.983	mRNA splicing	3.52E-03	2.031
6.80E-03	1.875	Pre-mRNA processing	1.12E-04	2.092
6.73E-06	1.663	Intracellular protein traffic	7.31E-06	1.622
9.93E-06	1.649	Cell cycle	1.67E-05	1.607

Significantly enriched Biological Processes and Molecular Functions in common for both gene lists have been identified by overlapping all terms that met the criteria as specified in Table 1.

**Table 3:** List of all unique and overlapping KRAB genes with significant enrichment of H3K9ac identified by HOMER in each of the conditions.

Arsenic (54 Genes)	Arsenic and Control (14 Genes)	Control (36 Genes)
2610008E11RIK	BC003267	D10627
2810021J22RIK	CTCF	ZBTB25
5730577I03RIK	FIZ1	ZBTB43
5730601F06RIK	REPIN1	ZBTB7A
6430526N21RIK	ZBTB6	ZFP109
BC027344	ZFP26	ZFP160
BC049807	ZFP27	ZFP191
D330038O06RIK	ZFP341	ZFP12
E430018J23RIK	ZFP386	ZFP2
GM16386	ZFP398	ZFP212
MYNN	ZFP579	ZFP236
PRDM15	ZFP61	ZFP316
PRDM2	ZFP668	ZFP334
RBAK	ZFP867	ZFP407
ZBTB17		ZFP41
ZBTB48		ZFP422
ZBTB5		ZFP513
ZFAT		ZFP568
ZFP157		ZFP57
ZFP184		ZFP580
ZFP202		ZFP592
ZFP248		ZFP605
ZFP260		ZFP606
ZFP263		ZFP612
ZFP273		ZFP644
ZFP28		ZFP709
ZFP282		ZFP780B
ZFP317		ZFP786
ZFP335		ZFP788
ZFP358		ZFP821
ZFP382		ZFP827
ZFP397		ZFP846
ZFP454		ZFP868
ZFP566		ZFP873
ZFP583		ZFX
ZFP60		ZKSCAN2
ZFP62		
ZFP623		
ZFP628		
ZFP637		
ZFP655		
ZFP672		
ZFP68		
ZFP697		
ZFP748		
ZFP763		
ZFP773		
ZFP787		
ZFP788		
ZFP810		
ZFP867		
ZFP9		
ZFP94		
ZKSCAN6		

Members of the KRAB box transcription factor family in arsenic and control gene sets (1757 and 1803 respectively, processed by DAVID for generation of Table 1) were identified in the corresponding output Functional Annotation tables. Note, that the lists in the table do not correspond to the lists presented on Table 1, where the output was according to the definitions of fold enrichment and Benjamini cut-off as described in the Methods.

### Memory and cognitive impairments in mice exposed to arsenic

We next examined the effect of arsenic exposure on memory and cognition in 6 months old mice. The mice were exposed to 100 μg/L arsenic in their drinking water for 2 weeks and arsenic exposure continued throughout the additional week of behavioral testing.

**Figure 3:**
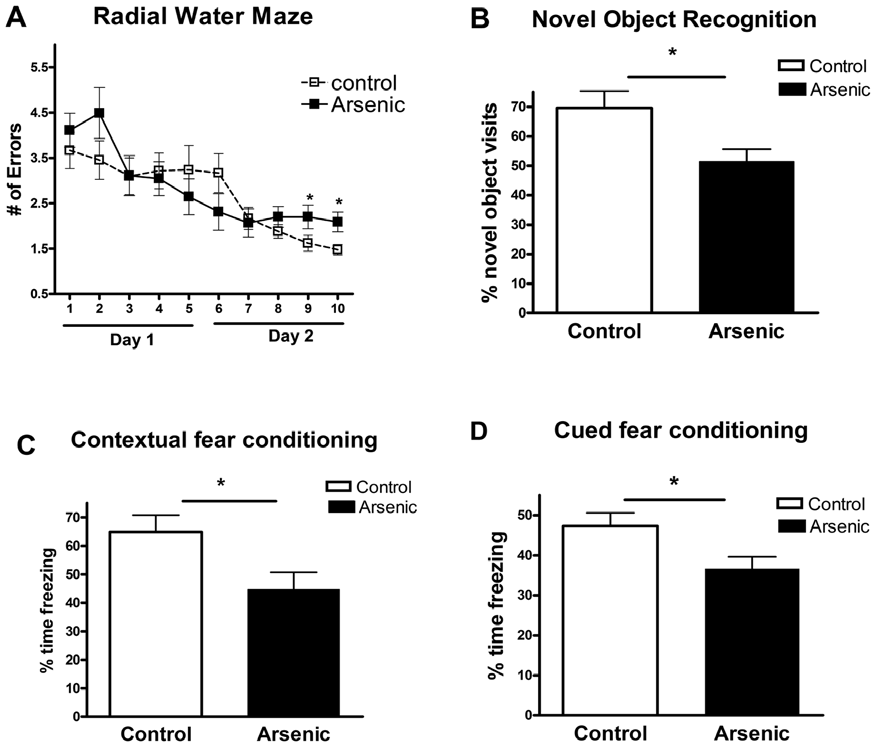
Arsenic treatment causes memory deficits in mice. Six months old C57BL6/J mice were exposed to 100 μg/L arsenic supplied in the drinking water for 3 weeks (N = 15). Control mice received plain water (N = 14). At the end of the treatment, arsenic treated and control mice were subjected to different behavior paradigms to assess cognitive performance. **A**. Radial water maze demonstrates that the spatial memory is affected by arsenic on the last two trial blocks. **B**. Novel object recognition shows a significant difference between arsenic and control group of mice. **C** and **D**. Arsenic treatment significantly impairs contextual (C) and cued fear conditioning (D). Data are presented as means ± SEM and analyzed by t-test. *, p<0.05.

We performed a battery of tests which included three separate behavioral paradigms (radial water maze (RWM), novel object recognition and contextual fear conditioning) to test cognitive changes due to arsenic treatment. These tasks are widely applied for evaluation of cognitive and memory changes associated with therapeutic treatments and/or evaluation of neurotoxicity in rodents, particularly in young adults. We utilized the RWM to assess spatial learning and working memory function. The RWM has a spatial complexity and performance measurement simplicity combined with the rapid learning and strong motivation observed in the Morris Water Maze without requiring foot shock or food deprivation as motivating factors. We chose to assess changes in long term recognition memory function independent of spatial learning strategies with the novel object recognition task. Furthermore, we utilized the contextual fear conditioning to test amygdala dependent associative learning and memory in association to the context and cued sound. We underscore, that the goal of our study was to determine changes in cognition associated with arsenic exposure and not to assess changes in locomotor functionality. However, since the RWM and novel object recognition tasks are dependent on locomotor function we included testing of locomotor function into the methodological design of our behavioral tasks. In the RWM me include a day of testing where we assess the ability of mice to find a visible platform. Here we determine the amount of time the animal takes to find a platform with a flag projecting 6 cm from the surface of the water, which evaluates the motivation and ability of the mice to swim. For the novel object recognition task, we first acclimate the animals to the behavioral paradigm (40 cm ×40 cm box) on day one of training. Thus we are able to measure the path length the animals take during the 5 min acclimation period to determine if the animals exhibit any dry land locomotor functionality alterations. We assayed impairments of spatial learning and working memory by Radial Water Maze (RWM) [21], long-term episodic memory by novel object recognition test, and possible amygdala-dependent associative memory impairment by fear conditioning paradigms. We found that during the RWM testing there was a significant difference between the two groups in the last two blocks of the test. To test the long term recognition memory, we used a simple protocol involving exploration of a pair of identical objects during the familiarization phase, and separated by 24 hours, a testing phase when the mice were presented with a familiar and a new object [22], [23]. Compared to control mice that spent more time exploring the novel object (visits to the new object were 69% of all visits), arsenic exposed mice exhibited significantly impaired novel object recognition performance (only 51% of all visits were visits to the new object, p<0.05, by Student's t test; Figure 3, panel B). We also applied a conditioning paradigm to assess associative learning: fear conditioning to a context and a cue (a combination of paradigms widely applied to examine the effects of pharmacological interventions on cognition) [24]. The data on Figure 3 demonstrate that compared to control mice, arsenic exposed mice displayed significantly shorter freezing periods in the contextual stage of fear conditioning, as well as in the cued phase of the test. Taken together, the data presented clearly demonstrate the negative effect of short term arsenic exposure on cognitive performance in adult mice.

### Arsenic induced biochemical and molecular alterations in adult mice

**Figure 4:**
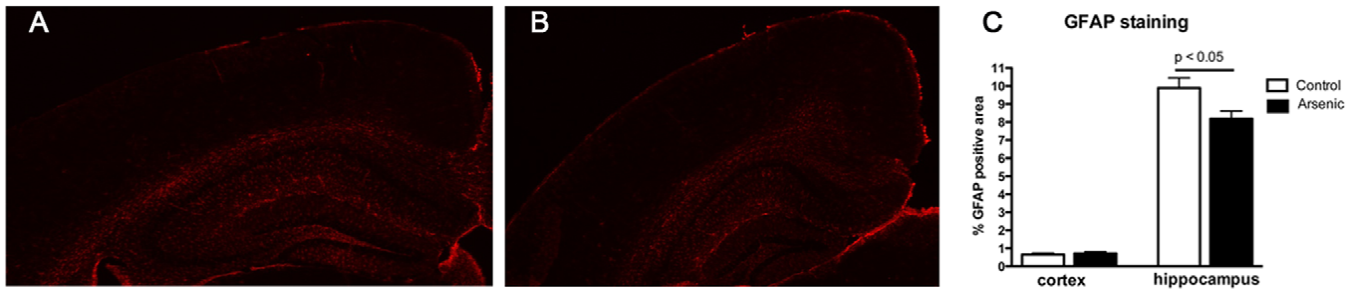
GFAP staining is decreased in mice exposed to arsenic. **A & B**. Staining on brain sections (A, control; B, arsenic exposed) using anti-GFAP antibody was used as a marker for astrocytes. Representative pictures illustrate the difference in GFAP staining in hippocampal area of control and arsenic treated mice. **C**. For graphical presentation, the intensity of staining in cortex and hippocampus was quantitated as explained in the methods. Analysis is by t-test. N = 7–8 mice per group.

**Figure 5:**
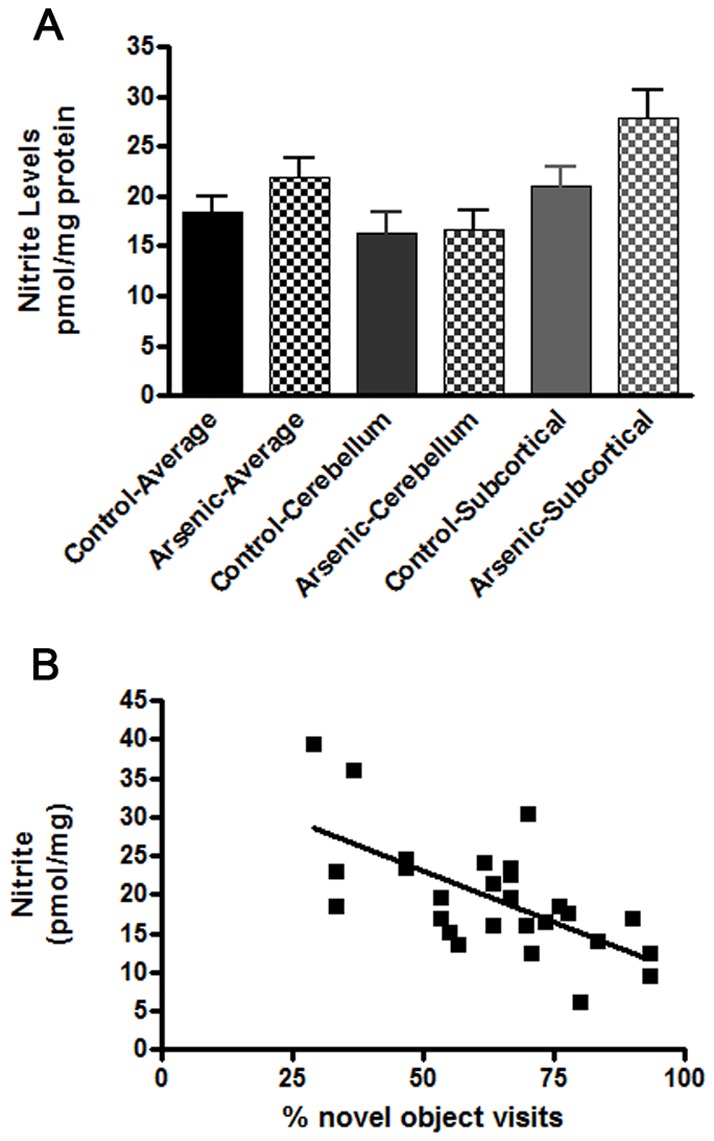
Nitrite levels in cerebellum and subcortical areas of mice exposed to arsenic. **A**. Nitrite levels were measured by tri-iodide based reductive chemiluminescence as in Curtis et al. [62] and presented as pmol/mg of protein. The average nitrite levels in cerebellum or subcortical tissue represent values relative to total nitrite levels in the brain. Although comparisons between control and arsenic samples (average total, cerebellum or cortical) indicate no significant changes, there is a strong trend towards an increase. **B**. A negative correlation between nitrite levels in arsenic treated mice and cognitive performance assayed by novel object recognition. The average nitrite concentration was correlated to the % novel object visits for control and arsenic mice (r^2^ = −0.4138, p<0.001). Control n = 14, arsenic n = 14.

We further evaluated morphological, biochemical and gene expression processes known to be altered by arsenic exposure and potentially involved in cognitive performance and memory formation. First, we measured the intensity of glial fibrillary acidic protein (GFAP) staining in the cortex and hippocampus of exposed and control mice. In some *in vitro* studies it has been demonstrated that neurotoxic effects of arsenic might be related to damages in astrocytes shown by cell viability, DNA damage and supported by morphological observations [25]–[27]. We have used staining for GFAP as an informative marker for number of astrocytes within well-defined brain structures [28], and found that there was a moderate, however, significant decrease in intensity of staining in the hippocampus of arsenic treated offspring (Figure 4). Considering the well-established role for astrocytes in supplying cholesterol and phospholipids to neurons (molecules, indispensable in maintaining synaptic plasticity and neuronal regeneration), our interpretation of these results is that, short duration environmentally relevant arsenic exposure in rodents does not initiate proliferation of astrocytes and might cause a decreased viability of astroglial cells. Since it has been shown that after toxic stimuli activation of NOX enzymes can induce damage of astrocytes (reviewed in [29], [30]) we measured the level of nitrites in the cerebelum and subcortical structures of exposed to arsenic and control mice. Our results show a trend towards increased nitrite levles, a sign of increased NOX enzyme activation after exposure to arsenic and in conjunction with the decreased GFAP staining. These data suggest a mechnaism of increased nitrite generation leading to astrocyte death. It is interesting to mention, that in some AD animal models inhibition of NOX enzymes and decreased levels of nitrites improved behavioral deficits [31]. Consistent with this we find a negative correlation between nitrite levels in mice epxosed to arsenic and their cognitve performance in novel object recognition test (Figure 5).

**Figure 6:**
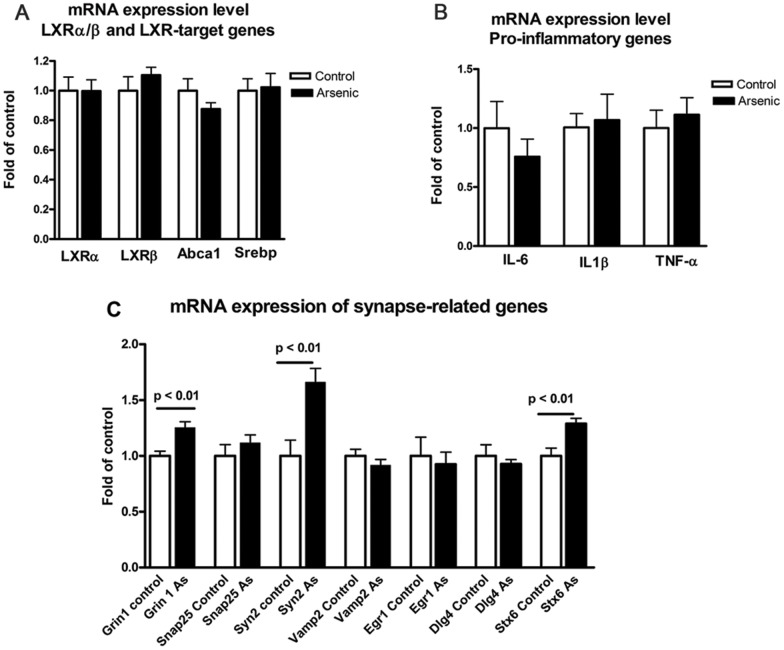
Effect of arsenic on gene expression level. Six months old C57BL6/J mice were exposed to 100 ppb arsenic supplied in the drinking water for 3 weeks and control mice received plain water. RNA was isolated from the cortices and mRNA expression level determined by RT-QPCR. **A**. Expression levels of LXRα/β and principal LXR-target genes are not changed by arsenic exposure. **B**. Exposure to arsenic does not change the expression level of pro-inflammatory genes. **C**. mRNA expression of selected synapse-related proteins in response to arsenic exposure. Abbreviations: Abca1 ATP-binding cassette, sub-family A, member 1 (Abca1), Sterol regulatory element binding transcription factor 1 (Srebp), Glutamate receptor, ionotropic, N-methyl D-aspartate 1 (Grin1), Discs, large homolog 4 (Dlg4), Phosphatidylinositol binding clathrin assembly protein (Picalm), Synatosomal-associated protein, 25 kDa (Snap25), synapsin II (Syn2), Vesicle- associated membrane protein 2 (Vamp2), Early growth response 1 (Egr1), Syntaxin 6 (Stx6), Nuclear receptor subfamily 1, group H, member 3 (LXRα), Nuclear receptor sub- family 1, group H, member 2 (LXRβ), Interleukin 6 (Il6), Interleukin 1 beta (Il1β), Tumor necrosis factor (Tnf), Cytochrome c oxidase subunit II (Cox-2) and Ribosomal protein, large, P0 (M36b4).

To further investigate possible impairment of brain cholesterol metabolism we compared the expression level of transcription factors LXRα and LXRβ and two of their principal target genes, Abca1 and Srebp by RT-QPCR, in control and exposed mice. There is a dual rationale to evaluate the expression levels of those genes: i) the role of LXRs and Abca1 in promoting memory formation and cognitive performance is now firmly established at least in animal models for Alzheimer's disease (review in [32]); ii) in an *in vitro* model it was demonstrated that expression level of LXR and some of their target genes is significantly inhibited by arsenic [33]. The graph in Figure 6, panel A demonstrates, however, that expression of LXRs, Abca1 and Srebp was unchanged in arsenic exposed mice. Considering the role of arsenic in the development of nonmalignant inflammatory lung disease, we also measured the expression level of pro inflammatory genes in brain tissue of exposed and control mice and similarly to cholesterol controlling genes did not find any difference. Lastly, we evaluated by RT-QPCR an array of synapse-related genes considered important for synaptic transmission, plasticity and synaptic vesicle transport. We found 3 of the genes – Glutamate receptor, ionotropic, N-methyl D-aspartate 1 (Grin1), Synapsin II (Syn2), and Syntaxin 6 (Stx6) up-regulated at significant levels. We did not find changes in the protein level by WB of any of those (data not shown).

## Discussion

Arsenic exposure at concentrations greater than the drinking water standard of 10 μg/L from both natural and man-made sources is a prevalent global issue. Epidemiological studies have linked low to moderate level of exposure to a host of disease states including cancer and cardiovascular disease in adults. In children, levels of exposure negatively correlate to IQ when measured by multiple matrixes (reviewed in [34]). While studies looking at toxic levels of arsenic in individuals demonstrated direct links to various diseases (except for cancer), and in most of the studies the results were adjusted for confounding factors such as length of exposure, diet, education, smoking, and levels of other water contaminants, a direct causality of low arsenic exposure to a certain disease has not been revealed.

To date most animal studies examining arsenic exposure and disease outcomes have focused on either prenatal exposure, or very high level exposure to arsenic (on the order of parts per million). These extreme levels are linked to a host of symptoms indicating toxicity, such as loss of weight and increased mortality, and do not reflect accurately effects of environmental levels of arsenic exposure in humans where such symptoms of toxicity are absent. Here, we report that by using environmentally relevant levels of arsenic exposure in adult mice we can explore disease outcomes and mechanisms associated with natural arsenic exposure in drinking water. Importantly, in our study with the low to moderate level used (100 μg/L), we did not find any evidence of overt toxicity or mortality and the weight of exposed adult animals was unchanged. It is clearly evident, however, that mice exposed to low levels of arsenic for a relatively short period of time demonstrate cognitive impairment in a battery of behavioral tests. A recent study with a rat model for prenatal exposure has demonstrated a poor performance in fear conditioning paradigm if animals were continuously exposed to environmentally relevant arsenic doses from gestation until 4 months of age. The methylation pattern of two genes considered important for memory formation – reelin (RELN) and protein phosphatase 1 (PP1), however, was unchanged [35]. Earlier and very recent studies reported by Luo et al. [12], [36] demonstrated that arsenic exposure, at doses almost 700 times higher than those used in our study, initiated a pronounced inhibitory effect on the expression of NR2A subunit of NMDA. NR2A is a nonobligatory subunit of the NMDA heteromeric complex, its incorporation increases after birth and similar to NR1A/Grin1 is important for Ca^2+^ influx and its downstream effects on Erk1/2 mediated signal transduction pathways. In our study, a poor cognitive performance after only 3 weeks of exposure was associated with a moderate decrease in GFAP staining, an increased mRNA expression level of Grin1, and a trend to an increased generation of nitrites and a negative correlation of those levels to performance of arsenic exposed mice in at least one of the behavioral tasks (Figure 5). Direct *in vivo* toxic effect of arsenic and metal mixtures on astrocytes with apoptotic cell death and increased ROS generation were demonstrated recently, and an increased Ca^2+^ release was suggested as an explanation [26]. Since NMDA receptor is directly involved in calcium homeostasis [37], sustained up-regulation of Grin1, and therefore a possible disruption of the homeostasis, instigated by arsenic exposure during the embryonic life may provide, at least in part, a molecular mechanism for arsenic neurotoxic effects. While the data from our study does not allow correlation of impaired learning in adult mice to changes in expression level and histone modifications, overall the results are urging an association that should be examined in the future with a goal to reveal genome-wide, and in an unbiased way, changes in expression of genes that may be critical for learning, memory and synaptic plasticity.

In recent years arsenic toxicity and its long term effects on human health have been linked to changes in the epigenome. Arsenic is the only in the group of toxic elements that causes changes in all three epigenetic marks – DNA methylation, histone modifications and expression of noncoding RNAs [16]. Although the results from different studies have not been consistent, changes in DNA methylation are usually linked to arsenic methylation, which is the normal, physiologically induced, pathway for arsenic detoxification in rodents and humans [34]. The sequestration of CH3 groups by arsenic in the detoxification pathway is considered, therefore, one possible molecular mechanism for global and gene-specific DNA hypomethylation, causing changes in the transcriptional activity of genes implicated in the risk for development of certain diseases. It is difficult to reconcile this hypothesis with the results of some *in vivo* studies [38], as well as results from epidemiological studies, where DNA hypermethylation has been reported. The methylation pattern and level of methylation on specific lysine residues in the N-terminal histone tails also change in response to arsenic exposure [39]. However, similar to the changes in DNA methylation pattern, a molecular mechanism explaining concurrent increased and decreased methylation of different lysine residues in the animal models, or humans has not been suggested [17].

The first report that arsenic exposure causes a severe reduction in acetylation of core histones was published in 1983 [40]. At this time no clear biological function was assigned to post-translational histone modification. The results with cultured Drosophila cells suggested that the effect of sodium arsenite may be mediated through the activation of heat shock proteins. More recently changes in histone H3 acetylation [41], and reduced acetylation of H4K16 residue [42] were demonstrated in arsenic treated cultured urothelial and endothelial cells. Elevated histone acetylation responsible for up-regulation of genes involved in apoptosis and stress response has also been reported. The increased global histone acetylation as a result of arsenic exposure in those [43], [44] and other [39] *in vitro* studies was ascribed to HDAC (histone deacetylase) inhibition. Globally reduced H3K9 acetylation was reported in peripheral mononuclear cells of a population exposed to arsenic in drinking water [18].

An animal model to test the health effects of *in utero* exposure to arsenic was developed in 2003 and demonstrated a sex specific tumor formation in adulthood [45]; aberrant gene expression profiles and alterations in hepatic DNA methylation were associated with hepatocellular carcinoma formation following *in utero* exposure to arsenic [46]. Most recently, and to a certain extent in contrast to the results from *in vitro* and animal studies, strong positive correlation between maternal blood arsenic and cord blood arsenic and global methylation of cord blood DNA in a sex specific manner was reported for a population exposed to high levels of arsenic in drinking water [47], [48].

An animal model to test the effects of prenatal arsenic exposure genome-wide on histone modifications has not been previously proposed. In our study, we embarked on massive parallel high-throughput sequencing of DNA from brain tissue of offspring born to mice exposed to human relevant arsenic concentrations in drinking water. We specifically evaluated the level of H3K9 acetylation for two main reasons:

- acetylation of H3K9 marks an open chromatin state and there is a correlation be- tween the enrichment of H3K9Ac in close proximity to the TSS and transcriptional activity of a given gene [49];
- although there has been only one published study so far addressing the acetylation status of specific lysine residue in the N-terminal tail of histone H4 (H4K12) using NGS technology [50], based on the previous work there is a consensus that inhibition of HDACs correlates to an improved cognitive performance and memory formation [51]–[53]. The results of our study demonstrate that prenatal exposure to human relevant doses of arsenic causes genome-wide hypo-acetylation at H3K9 in cortices and hippocampi of offspring. We found that fold change over the input and the height of peaks representing regions of H3K9Ac enrichment within certain distance from the TSS are lower in the sequenced ChIP libraries generated from brains of arsenic exposed offspring. It was reflected by the number of peaks/regions of enrichment subjected to analysis – 37,946 and 8,577 respectively. Surprisingly, a pairwise comparison of functional annotation tables generated by DAVID and based on the top 3000 regions (by HOMER score) with a distance to the nearest gene ≤1 kb, revealed clusters of GO terms significantly enriched and unique for each of the groups, as well as clusters that overlap (Tables 1 and 2). While it is difficult to speculate on the relative importance of those clusters for each of the conditions, and to what extent they represent the epigenetic reprogramming instigated by arsenic exposure during a critical developmental window, two of the clusters, significantly enriched in arsenic treated group – KRAB box containing zing finger repressor proteins and Zinc finger transcription factors, are of particular interest. We believe the relative enrichment of these proteins in brains of arsenic treated offspring and the molecular interactions between KRAB and several other nuclear proteins provide an explanation for the global hypo-acetylation induced by arsenic exposure. KRAB box containing proteins were discovered in 1991 [54] and are coded for by more than 400 genes [55]; together with the other zinc finger proteins present in the human genome, this group of proteins is the largest single family of transcriptional regulators in mammals [55], [56]. While there is a limited knowledge about gene-specific KRAB mediated transcriptional repression and the number of the genes known to be repressed by KRAB box containing transcription factors is small, the molecular details of this repression are fairly well understood [56]. Based on the results of our study and what is considered a generally understood function of KRAB proteins, we suggest a model for the effect of arsenic by assuming that following arsenic exposure significantly more KRAB containing proteins become available for downstream molecular interactions providing a molecular framework for activity of protein complexes critical for histone modifications including H3K9 acetylation. The formation of those multi-molecular complexes begins with binding of KRAB box containing zinc finger proteins (already bound to their corresponding DNA sequences) to KRAB associated protein (KAP1). Binding to KAP1 is an absolute requirement for KRAB containing proteins to mediate transcriptional repression [57]–[59]. KAP1-KRAB complexes provide a scaffold for recruitment and stepwise assembly of powerful corepressor complex containing isoforms of HP1 (heterochromatin protein 1), HDACs (histone deacetylases) and Setdb1 (SET-domain protein) that methylates H3K9. Once assembled this multimeric protein complex causes chromatin condensation and provides heterochromatin environment on a target promoter for gene silencing. Deacetylation of H3K9 by HDAC is a key step in this complex molecular interaction for heterochromatin formation which precedes the methylation of the same residue by Setdb1. H3K9Ac and H3K9Me are found in different genomic regions and have opposing roles in transcriptional regulation [60]. Therefore it is not surprising that the relative enrichment of H3K9Ac in the promoter regions of KRAB box containing transcription factors as a result of arsenic exposure (or some other, untested so far condition) coincides with otherwise global hypo-acetylation at the same histone residue genome-wide. While testing of our hypothesis is outside the scope of this study, further investigation into the function and targets of KRAB genes may lead to an increased understanding if and how subtle changes in epigenetic marks instigated by environmental factors may lead to pathological phenotypes later in life. More importantly, if future studies succeed to confirm that the model truly represents the effects of arsenic exposure on the epigenome during a sensitive window of development; this will be an example of environmental control of long term gene regulation in rodents and humans through chromatin remodeling and accessibility - two molecular processes, critical for transcriptional activation and gene silencing.

## Methods

### Reagents

All general purpose reagents, if not otherwise specified, were from Sigma, Invitrogen or Thermo Fisher Scientific.

### Mice and Arsenic exposure

All animal experiments were approved by the University of Pittsburgh Institutional Animal Care and Use Committee. Any-Maze software used for recording and analysis of behavioral tests was from Stoelting (Wood Dale, IL). Sodium arsenite water (100 μg/L) was prepared every other day. For behavioral testing 6 mo. old mice were provided with either 100 μg/L arsenic in spring water (arsenic exposed) or plain spring water (control) for 2 weeks. Arsenic exposure continued throughout the additional week of behavioral testing. For prenatal exposure female mice (8–9 weeks old) were provided with either arsenic in spring water (arsenic exposed) or plain spring water (control) for 1 week. Males were then added to the cage and removed 8 days after 1st introduction. Females continued under arsenic exposure for the duration of their pregnancy. Pups were collected within 24 hrs of birth, and cortices and hippocampi dissected. Tissue from each litter was then pooled for all further assays.

### Behavioral tests

#### Radial Arm Morris Water Maze (RWM)

Spatial cognition was assessed in a circular pool of water (diameter 122 cm, height 51 cm, temperature 21±1°C) using a modified version of the two-day 6 arm Radial arm water maze [21]. The ability to navigate to the goal, was measured using a clear acrylic circular platform (diameter 10 cm) submerged 1 cm below the water surface, with several distal visual cues. Spatial acquisition was assessed over a 2 day period. Each day consisted of 15 trials in which the goal platform was placed in a single arm; animals were lowered in different arms, and were allowed to explore the maze for 60 sec. If the platform was found within 60 sec the trial was stopped and the animal rested on the goal platform for 20 sec. If the animal failed to locate the hidden platform within 60 sec, it was led there by the experimenter where it remained for 20 sec. On day 1 of training a flag was placed on the platform during trials 1, 3, 5, 7, 9, and 11. During all other trials the platform remained hidden. Errors were defined as the animal entering an arm which did not contain the goal and the number of errors per trial was grouped into blocks of three trials.

#### Novel Object Recognition

Following testing in the RWM changes in long term memory was assessed utilizing a novel object recognition behavioral paradigm [22], [23]. On day 1 of testing mice were habituated to the behavioral arena (40 cm ×40 cm ×30 cm tall- white plastic box) for 5 min. For training, twenty-four hours following habituation mice were placed into the center of the arena with two similar objects and allowed to explore the objects for 30 total visits but no longer than 10 min. The two identical objects were made of weighted plastic to prevent movement and located in the east and west quadrant, spaced equidistant from the arena walls. Twenty-four hours following training one object was replaced with a novel object, object replaced was alternated for each mouse to avoid a side preference and mice were again placed into the arena and allowed to explore the objects for 30 total visits or 10 min. The definition of an exploratory visit was that the mouse was sniffing, climbing on, or touching the object or was a within 1 cm while facing an object. All testing took place during the light phase of the animals light/dark cycle, testing was recorded with ANY-maze video tracking software and the arena was cleaned with 70% alcohol between animals to eliminate olfactory cues. An increased percentage of visits exploring the novel object (number of novel object visits/total visits ×100) was considered an index of improved long term memory in this task.

#### Fear conditioning

Testing was performed according to a previously published protocol [24]. Fear conditioning chamber was purchased from Stoelting (Wood Dale, IL). Mice were conditioned by placing each subject into the enclosure and the sound-attenuating chamber door closed. The animal was in the enclosure for a total of 3 min. The first two minutes had no auditory or shock cue. The sound event began after two minutes and played for duration of 30 sec. The 0.7 Amp foot-shock event began after 2.5 min and lasted for 2 sec. The animal was allowed to reacclimatize to the testing enclosure for another 30 seconds. Testing was completed 30 sec after the shock. Contextual fear testing: Animals were tested 24 hours after conditioning. The enclosures and transporting procedures for testing were identical to those on the training day. There was no sound or shock event. Animals were tested for duration of 3 min and their freezing behavior was monitored by the ANY-maze software via the video camera in the sound-attenuating box. Freezing was used as a measure of learning in this task and is defined as the total lack of movement except for respiration.

#### Cued fear testing

Animals were tested 24 hours after being tested for contextual fear conditioning. The enclosures and transporting procedures for testing were identical to those on the training day. The context of the enclosure was changed by changing the patterns on the walls of the enclosure. The animals were tested for 5 min. A sound event was played 2 min after the start for duration of 3 min and the freezing behavior was recorded by the ANY-maze software.

### Tissue collection

Mice were anesthetized with Avertin (250 mg/kg, by intraperitoneal injection) and perfused transcardially with 20 ml of 0.1 M PBS (pH 7.4). Brains were rapidly removed and divided into hemispheres with one hemisphere being dropped fixed in 4% phosphate-buffered paraformaldehyde for 48 hours and then stored in 30% sucrose at 4°C. The cortex and hippocampus of the other hemisphere were dissected and snap frozen on dry ice. Frozen samples were stored at −80°C before processing.

### RNA processing and RT-QPCR

RNA from 20 mg of cortex purified using RNeasy spin columns (Qiagen, Valencia, CA) according to the manufacturer's protocol. For real time- quantitative PCR (RT-QPCR) first-strand cDNA was synthesized using Sprint RT Complete Random Hexamer strips (Clontech, Mountain View, CA) from 300 ng of total RNA. cDNA was diluted 5 fold before 2 μl were used in RT-QPCR reactions. RT- QPCR was carried out using standard conditions on ABI 7900 Real-Time PCR System. Relative expression levels were calculated by comparative ΔΔCt method with M36B as endogenous control gene.

### Histology and immunohistochemistry

All procedures were as reported previously [61]. Histoprep-embedded hemibrains were cut in the coronal plane into 30 μm sections and stored in a glycol-based cryoprotectant at −20°C until staining. Sections were selected 700 μm apart starting from a random section 150 μm caudal to the first appearance of the hippocampus. Free-floating sections were washed with PBS and blocked with 3% normal goat serum. Sections were then incubated in anti-GFAP antibody (1∶1000) at room temperature for 3 hrs before incubation with anti-rabbit 594-labeled secondary antibody (DI-1594, dilution 1∶250, Vector Laboratories) for 1 hr. Microscopic examination was performed using Nikon Eclipse 80i microscope. For analysis, staining in the cortex and hippocampus was defined as the percentage of area covered by GFAP using MetaMorph 7.0 software (Molecular Devices, Sunnyvale, CA).

### Measurement of Nitrite levels

Cerebellum and brain tissue from subcortical areas were finely minced and rinsed twice with PBS to completely remove erythrocytes. The tissue was homogenized and suspended in Kreb-Henseleit buffer at a ratio 4mg tissue per mL buffer. In a closed chamber 0–21% O2 and 5% CO2 balanced with N2 gas mixtures (Matheson Gas, Pittsburgh, Pa) were passed over the homogenate to deoxygenated it. Nitrite was measured after tri-iodide (I_3_^−^) based reductive chemiluminescence in a vessel connected inline to a Nitric Oxide Analyzer (Sievers), as in our previous studies [62]. Samples were separated into two aliquots and left untreated or treated with acidified sulfanilamide (16% in 2 M HCl). Each aliquot was injected into I_3_^−^ and the area under the curve measured and concentration quantified using a standard curve of known nitrite concentrations. The concentration of nitrite was the difference between the aliquot left untreated and that treated with acidified sulfanilamide alone.

### Western Blot

Frozen cortices were homogenized in tissue homogenization buffer (250 mM sucrose, 20 mM Tris base, 1 mM EDTA, 1 mM EGTA, 1 ml per 100 mg tissue) and protease inhibitors (10 μg/ml leupeptin, 10 μg/ml aprotinin, and 10 μg/ml AEBSF [4-(2-aminoethyl)benzenesulfonyl fluoride]) using a glass Dounce. Homogenate was spun at 16,000× *g* for 10 min, and the supernatant removed. The remaining pellet was resuspended with RIPA buffer in the presence of protease inhibitors, sonicated for 15 s, and then spun again at 16,000× *g* for 10 min. Concentration of each sample was determined in Bradford assay by comparison to bovine serum albumin standard curve using linear regression analysis. For WB analysis, extracts containing 30 μg of total protein were mixed with Tris/glycine loading buffer, loaded, and electrophoresed on 10% Tris/glycine gels. Gels were transferred to nitrocellulose membranes, incubated with the respective primary antibodies followed by secondary antibodies conjugated to horseradish peroxidase, and processed for visualization by enhanced chemiluminescence Plus-ECL (PerkinElmer). The relative intensities of the bands were quantified by densitometry (ImageQuant, version 5.2; GE Healthcare).

Even though small differences in mRNA levels measured by RT-QPCR may not result in detectable differences at the protein level, we performed WB analysis of proteins which demonstrated significant changes in mRNA expression level; Stx6 was detected using monoclonal antibody, ab12370 (Abcam), Grin1 was detected using polycolnal antibody, ab77264 (Abcam) and Syn2 was detected using polyclonal antibody ab62737 (Abcam). We measured the protein levels of HDAC1 in the cortex of adult mice to determine if an increased protein level of this histone deacetylase is a consequence of arsenic exposure. HDAC1 was detected using polyclonal antibody sc-7872 (Santa Cruz Biotechnology), and no difference in protein level was detected (Data not shown). β-Actin was used as a loading control for all WBs and detected with monoclonal antibody, A5441 (Sigma-Aldrich).

### Chromatin immunoprecipitation

Chromatin for ChIP was prepared as described previously [63] with some modifications. Chromatin from a pool of 6–8 newborn mouse cortices was prepared by cross-linking with 2% formaldehyde in PBS followed by nuclear extraction. Chromatin was sheared to fragments of 200–500 bp by sonication with 3 pulses of 15 sec at 40 amplitude, and 3 pulses of 15 sec at 50 amplitude. Lysates were cleared by centrifugation, and supernatant was used for immunoprecipitation. ChIP for H3K9Ac histone modifications was performed on 100 μg of chromatin as described before [64] with ab10812 antibody against acetylated H3K9 (Abcam, Cambridge, MA). The final DNA pellet was resuspended in 50 μl of TE buffer and used for validation by RT-QPCR and high throughput sequencing. Validation of ChIP was performed by measuring the enrichment of select promoter regions using Power SYBR Green PCR Master mix and the ABI7900HT instrument (Applied Biosystems, Foster City, Ca). Analysis was performed by the standard curve method and % input was calculated for each promoter region. For comparison glucagon (Gcg) and tubulin (Tubb3) proximal promoters were used as negative and positive controls.

### Generation of ChIP Libraries and sequencing

Libraries for multiplex sequencing were generated using the NEB Next^TM^ ChIP-Seq DNA Sample Prep Reagent Set 1 (NEB E6200S/L, Ipswich, MA) according to the manufacturer's protocol with 40 μl of IP and 10 μl of Input DNA in a series of reactions. Library fragments were purified by incubation with Agencourt Ampure XP Beads, with two 75% ethanol washes. DNA fragments were run on a 2% agarose gel for size selection. Gel slices from the 200±25 bp range were excised, DNA purified and eluted with 38 μl EB. Final library amplification was performed by PCR on the size selected fragments using a common forward primer and an indexed reverse primer. The sequences of the primers for each sample are presented on Table 4. The PCR reaction was cleaned up using Qiagen's mini-elute PCR purification kit and before sequencing, the libraries were validated by Agilent Technologies 2100 Bioanalyzer to check the size, purity, and concentration of the sample. Samples were sequenced to 50 bp on an Illumina hiSeq2000. The first 36 bp of the sequences were then aligned to the mouse genome (NCBI Build 37, mm9) using the unique best alignment for reads, allowing up to 3 mismatches (Bowtie parameters -k 1 -m 1 –best –strata; Bowtie, http://bowtie-bio.sourceforge.net, [65], [66]). This produced approx. 29 million reads for control H3K9ac, 28M reads for control input, 24 M reads for arsenic-exposed H3K9ac, and 62 million reads for arsenic input samples. Enrichment was called using HOMER in histone mode with an FDR cutoff of 0.001. The fragment lengths were estimated using data from previous quality checks using an Agilent bioanalyzer, i.e., the mode of the libraries' length distribution's less the 120 bp for truSeq adapters. Regions of H3K9ac enrichment were associated with genes by identifying any overlapping genes, the nearest transcription start site (TSS) and any other TSS with 1.5x of the distance of the closest TSS. More stringent distance limits were then obtained by filtering this master list. The HOMER “Fold-Change-vs-Control” values were used as the scores of the regions. Profiles indicating coverage by ChIP (or input tracks) were made by extending aligned reads to the mean insert length, then counting the coverage at all positions in the genome. These profiles were then scaled to reads per million units for inclusion in figures. Data will be deposited in ArrayExpress and in the final version an accession number will be provided.

**Table 4:** Primers used for ChIP library generation.

	Primer sequence
Forward primer	AATGATACGGCGACCACCGAGATCTACACTCTTTCCCTACACGACGCTCTTCCGATC*T
**Sample/Reverse primer**	
Control Input	CAAGCAGAAGACGGCATACGAGATACATCGGTGACTGGAGTTCAGACGTGTGCTCTTCCGATC*T
Control IP	CAAGCAGAAGACGGCATACGAGATGATCTGGTGACTGGAGTTCAGACGTGTGCTCTTCCGATC*T
Arsenic Input	CAAGCAGAAGACGGCATACGAGATTGGTCAGTGACTGGAGTTCAGACGTGTGCTCTTCCGATC*T
Arsenic IP	CAAGCAGAAGACGGCATACGAGATTACAAGGTGACTGGAGTTCAGACGTGTGCTCTTCCGATC*T

### Gene ontology analysis

To identify biological processes in which the submitted genes were involved PANTHER analysis was performed using the Database for Annotation, Visualization and Integrated Discovery (DAVID, version 6.7; [20] with mm9 RefSeq mRNA annotations and the entire mouse genome as a background model. The fold enrichment score in the tables is provided by DAVID as in the functional annotation charts, and is defined as % genes in a given class/% genes in genome in the same class. Clusters were considered significant at Benjamini factor ≤0.05 and fold enrichment ≥1.5. Non informative classifications (such as “Other RNA-binding protein”) were removed. The GO analysis was performed for the top 3000 regions with a distance to the nearest gene ≤1 kb.

### Statistical Analysis

All results are reported as means ± SEM. Statistical significance of differences between mean scores during acquisition phase of training in the RWM were assessed with two-way repeated-measures ANOVA (general linear model/repeated-measures ANOVA) and Student t-test for comparisons trial block number as sources of variation. The data for contextual and cued fear conditioning and for novel object recognition were analyzed by Student t-test. All statistical analyses were performed in Prism (Graph Pad, version 4.0), or SPSS (IMB, version 19), and differences were considered significant where p≤0.05.
